# Operation for Extensive Symblepharon

**Published:** 1884-10

**Authors:** R. O. Cotter

**Affiliations:** Assistant to Chair of Eye, Ear and Throat Diseases, Atlanta Medical College; Atlanta, Ga.


### OPERATION FOR EXTENSIVE SYMBLEPHARON.

BY R. O. COTTER, M. D.,

*Assistant to Chair of Eye, Ear and Throat Diseases, Atlanta Medical College.*

Dave W., colored, aged 40, applied for treatment sometime in 1883, mainly for the relief of a severe iritis in his left eye, the result of blow from a hair brush received in a fracas with another negro. The injury had been received some weeks before I saw him, and the eye presented the appearance of having been considerably bruised and inflamed. The conjunctiva of the whole lower lid, and of the lower half of the ball had become firmly united by adhesions. This condition caused him considerable pain upon the slightest attempt to move the eye ball. The adherent lid had also extended so far upward on the cornea as to almost entirely cover the pupil. All movement of the ball, except in a downward direction, was prevented. Attention was first directed to the relief of the intense iritis, as adhesions had already taken place between the lens and iris, and the pupil thus bound down and also nearly all covered by the adherent lid, his vision was practically lost for the time being. The adhesions of the iris and lens were broken up by a 3 gr. to the ℥ solution of sulph. atropia, applied several times daily for several days, and the iritis also gradually relieved by the free use of the atropia solution and blisters applied behind the ear and to the temple. After the iritis had been relieved he was told that an operation would be necessary to relieve him of the pain and inconvenience caused by the adhesion between the lid and ball, but he would not then consent to an operation.

After some months (May 17th, 1884) he again presented himself and signified his desire to have the operation made, as his eye was practically useless to him, on account of the lack of motion of the ball, and partially obstructed pupil.

Without using any anæsthetic (although the operation was quite painful) the lid and ball were separated as carefully as possible and the adherent lid shaved off the cornea by means of a Beer's cataract knife.

As it was necessary to preserve as much as possible of the conjunctiva either of the lid or the ball, I endeavored to save as much as practicable of that of the ball in order to leave as little as possible of the two raw surfaces in opposition.

After separating the two raw surfaces what little free conjunctiva of the ball that could be obtained was carefully stitched together by very fine sutures, and cold water dressings applied for two or three days; vaseline was also applied to the raw surfaces. Some slight tendency towards reunion was broken up by use of a probe. He was also advised to frequently rotate the eye ball in various directions to aid in preventing the reunion, as this is the difficulty feared after these operations, especially in extensive cases, as this one was. The operation proved perfectly successful and the patient now has perfect movement of his eye ball and has normal vision.

